# Accessing the discriminatory performance of FRAIL-NH in two-class and three-class frailty and examining its agreement with the frailty index among nursing home residents in mainland China

**DOI:** 10.1186/s12877-019-1314-9

**Published:** 2019-10-30

**Authors:** Feng Ge, Weiwei Liu, Minhui Liu, Siyuan Tang, Yongjin Lu, Tianxue Hou

**Affiliations:** 1 Xiangya Nursing School, Central South University, Changsha, Hunan China; 2 Center for Innovative Care in Aging, Johns Hopkins University School of Nursing, Baltimore, MD USA

**Keywords:** Discriminatory performance, Agreement, FRAIL-NH, Frailty index, Nursing homes

## Abstract

**Background:**

FRAIL-NH has been commonly used to assess frailty in nursing home residents and validated in many ethnic populations; however, it has not been validated in mainland China, where such an assessment tool is lacking. This study aimed to (1) assess the discriminatory performance of FRAIL-NH in two-class frailty (non-frail+ pre-frail vs. frail) and three-class frailty (non-frail vs. pre-frail vs. frail), based on the Frailty Index (FI), (2) determine the appropriate cutoff points for FRAIL-NH that distinguish two-class and three-class frailty, and (3) examine the agreement in classification between FRAIL-NH and FI.

**Methods:**

A cross-sectional study of 302 residents aged 60 years or older from six nursing homes in Changsha was conducted. The FRAIL-NH scale and 34-item FI were used to measure frailty. Two-way and three-way receiver operating characteristic (ROC) curves were used to estimate the performance of FRAIL-NH. Cohen’s Kappa statistics were used to examine the agreement between these two measures.

**Results:**

The agreement between FRAIL-NH and FI ranged from 0.33 to 0.55. Regardless of what FI cutoff points were based on, the volume under the ROC surface (VUS) for FRAIL-NH from the three-way ROC were higher than the VUS of a useless test (1/6), and the area under the ROC curve (AUC) for FRAIL-NH from the two-way ROC were higher than the clinically meaningless value (0.5). When using FI cutoff points of 0.20 for pre-frail and 0.45 for frail, FRAIL-NH cutoff points of 1 and 9 in classifying three-class frailty had the highest VUS and the largest correct classification rates. Whichever FI was chosen, the performance of FRAIL-NH in distinguishing between pre-frailty and frailty, and between non-frailty and pre-frailty was equivalent. According to FRAIL-NH, the proportion of individuals with frailty misclassified as pre-frailty was higher than that of individuals with non-frailty misclassified as pre-frailty.

**Conclusion:**

FRAIL-NH can be used as a preliminary frailty screening tool in nursing homes in mainland China. FI should be further used especially for those classified as pre-frailty by FRAIL-NH. It is not advisable to simply combine adjacent two classes of FRAIL-NH to create a new frailty variable in research settings.

## Background

The management of frailty in nursing home residents is a significant problem in China. First, the prevalence of frailty measured by the Tilburg Frailty Indicator (TFI) and the Physical Frailty Phenotype (PPF) from cross-sectional studies is high in nursing home residents in China (55.1–55.7%), and it is expected to increase continuously in the next decades [1, 2]. The increasing prevalence of frailty in China can make frailty management more difficult in older adults. Second, many nursing home residents in China live a sedentary lifestyle, which may contribute to further declines in physical function and an acceleration of the frailty process [3]. Third, the lack of frailty measures specific for nursing home residents in China has exacerbated this problem because a substantial proportion of pre-frail or frail older adults has not been identified and thus could not receive timely and appropriate intervention. Adopting an effective frailty assessment tool specific to nursing home residents is a crucial first step for managing frailty in China.

Frailty Index (FI) and PPF are well-recognized and commonly used frailty assessment tools in nursing home settings [4]; however, they are not always feasible in routine practice in Chinese nursing homes because they are complex and time-consuming to use and are difficult to operate [5–8]. Other frailty assessment tools (e.g., TFI [9], Clinical Frailty Scale [10]) were mainly developed for community-dwellers and inpatients and were inappropriate for nursing home residents. Thus, the true prevalence of frailty in Chinese nursing homes could not be accurately assessed by these tools. Kaehr et al. developed the FRAIL-NH scale specific to nursing home residents after combining the core characteristics of FI and PFF [11, 12]. FRAIL-NH is a short, easy-to-administer and valid frailty assessment tool with high predictive ability of adverse outcomes [6, 11, 13]. It has been validated in many countries around the world, including the U.S. [11], France [6], Spain [8], Australia [7] and Hong Kong in China [13]. However, to the best of our knowledge, it has not been validated in mainland China.

The clinical and research utility of FRAIL-NH in assessing frailty needs to be evaluated adequately before its ultimate use in mainland China as a screening tool. First, it is essential to determine the discriminative ability of FRAIL-NH in classifying different levels of frailty and the corresponding cutoff points. ROC curve analysis is a valid method to estimate the discriminatory ability of screening tools and to determine their best cutoff points that can directly aid diagnostic decisions [14]. Different FRAIL-NH cutoff points have been established in countries such as Australia and Spain [7, 8, 11, 13]; however, these cutoff points might be inappropriate for nursing home older adults in mainland China because the cutoff points vary in different cultures and ethnicities [15]. Determining the appropriate FRAIL-NH cutoff points offers a simple and accurate way to identify older people who are at risk of frailty and provide timely intervention. Besides, the performance of FRAIL-NH in discriminating non-frailty, pre-frailty and frailty has not been extensively investigated.

Second, as another useful metrics for clinical utility, the agreement of FRAIL-NH measured by kappa statistics needs to be examined with an alternative measure, usually an established gold standard. FI, which captures the multidimensionality of frailty with stronger predictive power for adverse outcomes [16, 17], has often been considered the gold standard of frailty diagnosis in clinical and research practice [18–20]. Many studies have compared the agreement between PPF and FI [21–24], but the agreement between the FRAIL-NH and FI has not been examined.

Third, the research utility of FRAIL-NH also needs to be evaluated. It is a common practice that researchers may create a two-class frailty variable (non-frail+ pre-frail vs. frail) by simply combining the adjacent categories of three-class frailty (non-frail, pre-frail vs. frail) due to statistical consideration such as a small sample size in a particular category. This raises methodological concerns as cutoff points for two-class and three-class frailty might be different.

Taken all together, this study sought to (1) assess the discriminatory performance of FRAIL-NH in two-class frailty (non-frail+ pre-frail vs. frail) and three-class frailty (non-frail vs. pre-frail vs. frail), based on FI; (2) determine the appropriate cutoff points for FRAIL-NH that distinguishes two-class and three-class frailty; and (3) examine the agreement in classification between FRAIL-NH and FI.

## Methods

### Study design, setting, and sample

A cross-sectional study was conducted in six nursing homes in Changsha, a provincial city in China between July and August 2018. A total of 302 nursing home residents were included in this study based on the inclusion and exclusion criteria, and the completeness of data. Details about the study design and process were described elsewhere [25]. The study was approved by the Nursing and Behavioral Medicine Research Ethics Committee of Central South University Xiangya Nursing School (IRB approval number: 2018012).

### Measurements

#### Frail-NH

The FRAIL-NH includes seven items: fatigue, resistance, ambulation, incontinence, weight loss, nutritional approach, and help with dressing [11, 12]. Possible total scores ranged from 0 (the best state) to 14 (the worst state). Detailed information about the FRAIL-NH is presented in Additional file 1: Table S1.

#### Frailty index

The FI is a count of impairments and illnesses, collectively known as deficits [10]. Each deficit of FI was coded 0 or 1 representing absence or presence, respectively. At least 30 age-related health deficits should be included to calculate a FI [26]. We developed a 34-item FI based on previous studies [5, 13, 26, 27]. The FI score was defined as the ratio between existing deficits and the number of evaluated deficits. Thus, the FI ranged from 0 to 1 (no deficit present, to all deficits present). The detailed information of the FI items and coding are shown in Additional file 1: Table S2.

To detect the severity of frailty and the agreement in classification between both measures, FI, which is a continuous score, was categorized based on cutoff points proposed in studies by Song et al. [28], Hoover et al. [5, 27], and Saum et al. [29], because they have widely been applied.

#### Covariates

Sociodemographic data including age, gender, educational level, marital status, weight, and height, were collected. Participants’ education levels were categorized into three groups: uneducated/primary, secondary, and university. Marital status was categorized into two groups: never married/widowed or divorced, and married. Body mass index (BMI) was calculated as weight divided by height squared (kg/m^2^).

### Statistical analyses

Both two-class and three-class frailty was used when examining the agreement and ROC curves. Based on Song et al. [28], we transformed a FI score into a three-class variable: FI ≤ 0.08 is non-frail; 0.08 < FI < 0.25 is pre-frail; FI ≥ 0.25 is frail. Based on Saum et al. [29], we also transformed a FI score into another three-class variable: FI ≤ 0.20 (non-frail), 0.20 < FI <0.45 (pre-frail), and FI ≥ 0.45 (frail). Additionally, the FI score was originally a four-class variable (non-frail, pre-frail, frail, most frail) in Hoover’s study [5, 27]. We used this variable to construct three-class frailty by collapsing the frail and most frail groups according to previous studies [5]. Therefore, a score of FI ≤ 0.10 was considered “non-frail,” a score of 0.10 < FI ≤ 0.21 was considered “pre-frail,” and a score of 0.21 < FI ≤ 1 was considered “frail.” Based on these cutoff points, the non-frail group and pre-frail group were combined again, and a FI cutoff points of 0.25, 0.21, and 0.45 were used to create two-class frailty according to Song et al. [28], Hoover et al. [5, 27], and Saum et al. [29], respectively.

Two-way ROC analyses were used to evaluate the performance of FRAIL-NH for two-class frailty and to determine the best cutoff points of FRAIL-NH when distinguishing two-class frailty. The two-dimensional ROC curve was constructed with sensitivity and specificity. The best cutoff points of FRAIL-NH can be obtained by examining the FRAIL-NH score that maximized sensitivity and specificity in differentiating two-class frailty based on the FI. The areas under the ROC curves (AUC) were computed to estimate the relative classification ability of FRAIL-NH for two-class frailty. An AUC of greater than 0.9, 0.7–0.9, or 0.5–0.7 represented high, moderate, and low diagnostic accuracy for frailty, respectively [30].

Three-way ROC analysis is a valid method to assess the performance of the test simultaneously in all classes [14, 31]. In this study, three-way ROC analyses were applied to determine the corresponding aspects of FRAIL-NH for three-class frailty, such as the discriminatory performance and the best cutoff points. Through the three-way ROC analysis, a three-dimensional ROC surface—where its coordinates represent three correct classification rates (CCR) obtained for each class (X = CCR_1_, Y = CCR_2_, Z = CCR_3_)—was described. The best pair of cutoff points on the ROC surface can be achieved by the pair that corresponds to the coordinate on the ROC surface with minimized squared distance to the perfect classification coordinates (1, 1, 1), where the CCRs of the three classes were 100% and the discriminative ability of the FRAIL-NH was the largest [14]. Of this pair of cutoff points, one was employed to distinguish between “non-frail” and “pre-frail” and the other for “pre-frail” and “frail.” The volume under the ROC surface (VUS), an extension of the AUC, is commonly used as an overall performance index of the discriminative accuracy [14, 31]. The VUS varies from 1/6 (useless classification/classifying by chance alone) to 1 (perfect classification), respectively [31]. Therefore, only when the VUS was higher than 1/6 = 0.17 was the diagnostic test considered “good” [32]. The parametric method was used to calculate VUS, its standard error (SE) and confidence interval (CI) when each class follows a normal distribution. Otherwise, the nonparametric method was applied. Besides, to provide further insight into the differences in the three classes, pair-wise comparisons via two-way ROC curves were also performed in a post hoc test to examine the ability of the FRAIL-NH to discriminate between each pair of frailty classes. The area under the three pairwise ROC curves was calculated for the curve corresponding to the comparison between non-frailty vs. pre-frailty, between non-frailty vs. frailty, and between pre-frailty vs. frailty.

The MATLAB 2016a program (MathWorks Natick, MA) and R 3.5.3 software were used for the three-way ROC analysis. The package DiagTest3grp developed by Luo and Xiong [14] in the R program was used to calculate VUS, SE, CIs, the Youden Index and to determine the best cutoff points. The code developed in MATLAB was used to draw the ROC surface and to perform the two-way ROC analyses as a post hoc test.

Of the total 302 participants, 3.3% (*n* = 10) had missed data on the Mini-Mental State Examination (MMSE); 3.3% (n = 10) had missed data on the Mini Nutritional Assessment Short Form (MNA-SF). Missing data were mainly because residents with cognitive impairment were not able to respond to specific items and residents with hand problems could not write to complete some MMSE items. No data were missing for the FRAIL-NH. The mean substitution was used to handle missing data. The mean score for the remaining items was imputed for the missing items. IBM SPSS Statistics version 18.0 (IBM Corp., Armonk, NY) was used for descriptive statistics and Cohen’s kappa statistics. Descriptive statistics were reported as means ± standard deviation (SD) for continuous variables or percentages for categorical variables. Cohen’s kappa statistics were used to measure the agreement in classification between FRAIL-NH and FI. Agreement was identified as poor for a Kappa coefficient of ≤0.20, fair for 0.21–0.40, moderate for 0.41–0.60, good for 0.61–0.80, and excellent for 0.81–1.00 [33].

## Results

Table 1 shows the sociodemographic characteristics of 302 nursing home older adults. They were aged between 60 and 100 years (mean age 82.71 ± 8.49); 71.2% of them were female. The majority were never married/divorced/widowed (77.5%), uneducated or with primary education (48.0%), and within normal BMI (61.6%). Mean FRAIL-NH and FI scores were 4.11 ± 3.65, and 0.27 ± 0.11, respectively.

**Table 1:** Sociodemographic and health characteristics of the study sample

Variables	Mean ± SD / n (%)
Age, years	82.71 ± 8.49
Age group	
60–79 years	87 (28.8)
80–100 years	215 (71.2)
Sex	
Male	87 (28.8)
Female	215 (71.2)
Marital status	
Never Married/Divorced/Widowed	234 (77.5)
Married	68 (22.5)
Education level	
Uneducated/Primary	145 (48.0)
Secondary	118 (39.1)
University	39 (12.9)
BMI, Kg/m^2^	22.94 ± 5.45
BMI	
Normal	186 (61.6)
Overweight	81 (26.8)
Obesity	35 (11.6)
FI, 0–1	0.27 ± 0.11
FRAIL-NH, 0–14	4.11 ± 3.65

*BMI* Body mass index (weight/height^2^, kg/m^2^); *FI* Frailty Index; *SD* Standard deviation

Table 2 shows the cutoff points of FRAIL-NH to classify two-class frailty based on FI. The two-way ROC curve analysis showed that the AUC for the FRAIL-NH was 0.87 (95% CI: 0.84–0.91), 0.86 (95% CI: 0.82–0.90), and 0.93 (95% CI: 0.89–0.97) when using a FI cutoff point of 0.25, 0.21, and 0.45, respectively. The optimal cutoff points of FRAIL-NH in classifying two-way frailty were 4 (66.1% sensitivity, 90.2% specificity), 2 (87.6% sensitivity, 66.3% specificity), 8 (94.1% sensitivity, 82.8% specificity) when using a FI cutoff point of 0.25, 0.21, and 0.45, respectively, according to the maximum principle of Youden’s Index.

**Table 2:** The cutoff points of FRAIL-NH to classify two-class frailty (Non-frail + Pre-frail vs Frail) based on FI

Frailty class	FI	FRAIL-NH				
	Cutoff points	Cutoff points	AUC (95% CI)	Sensitivity (%)	Specificity (%)	Youden Index
	0.25	4	0.87 (0.84–0.91)	66.1	90.2	0.56
Two-class	0.21	2	0.86 (0.82–0.90)	87.6	66.3	0.54
	0.45	8	0.93 (0.89–0.97)	94.1	82.8	0.77

*FI* Frailty Index; *CI* confidence interval; *AUC* the area under the curve

Table 3 presents the cutoff points of FRAIL-NH to classify three-class frailty based on FI. The three-way ROC curve analysis showed that the VUS for the FRAIL-NH was 0.68 (95% CI: 0.57–0.76), 0.62 (95% CI: 0.55–0.72), and 0.76 (95% CI: 0.70–0.81) according to the cutoff points of the FI developed by Song et al. [28], Hoover et al. [5, 27] and Saum et al. [29], respectively. The optimal pair of FRAIL-NH cutoff points for three-class frailty were 0 and 4 (FRAIL-NH = 0 for non-frail, 0 < FRAIL-NH ≤ 4 for pre-frail, FRAIL-NH > 4 for frail) based on the cutoff points of the FI suggested by Song et al. [28] or Hoover et al. [5, 27], and 1 and 9 (0 ≤ FRAIL-NH ≤ 1 for non-frail, 1 < FRAIL-NH ≤ 9 for pre-frail, FRAIL-NH > 9 for frail) based on the cutoff points of the FI suggested by Saum et al. [29]. Accordingly, the CCR corresponding to three classes was as follows: CCR_1_ = 80.0%, CCR_2_ = 66.1%, CCR_3_ = 66.1% based on the cutoff points of the FI suggested by Song et al. [28]; CCR_1_ = 65.0%, CCR_2_ = 67.9%, CCR_3_ = 60.7% according to the cutoff points of the FI developed by Hoover et al. [5, 27]; and CCR_1_ = 70.9%, CCR_2_ = 64.3%, CCR_3_ = 88.2% according to the cutoff points of the FI developed by Saum et al. [29].

**Table 3:** The cutoff points of FRAIL-NH to classify three-class frailty (Non-frail vs Pre-frail vs Frail) based on FI

Frailty class	FI	FRAIL-NH					
	Cutoff points	Cutoff points	VUS (95% CI)	CCR_1_(%)	CCR_2_ (%)	CCR_3_ (%)	Youden Index
Three-class	0.08, 0.25	0, 4	0.68 (0.57–0.76)	80.0	66.1	66.1	0.58
	0.10, 0.21	0, 4	0.62 (0.55–0.72)	65.0	67.9	60.7	0.56
	0.20, 0.45	1, 9	0.76 (0.70–0.81)	70.9	64.3	88.2	0.69

*FI* Frailty Index; *VUS* volume under the ROC surface; *CI* confidence interval; *CCR* correct classification rate

**Fig.1:**
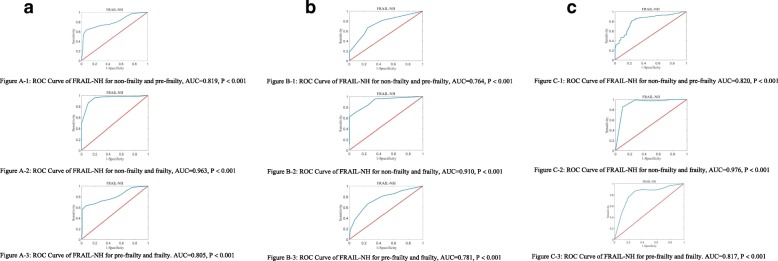
**a** Receiver operating characteristic (ROC) curves of FRAIL-NH for pairwise comparisons (The gold standard FI: FI ≤ 0.08 for non-frailty, 0.08 < FI <0.25 for pre-frailty, and FI ≥ 0.25 for frailty; FRAIL-NH: FRAIL-NH = 0 for non-frailty, 0 < FRAIL-NH ≤ 4 for pre-frailty, and FRAIL-NH > 4 for frailty). 1: ROC Curve of FRAIL-NH for non-frailty and pre-frailty, AUC = 0.819, *P* < 0.001. 2: ROC Curve of FRAIL-NH for non-frailty and frailty, AUC = 0.963, *P* < 0.001. 3: ROC Curve of FRAIL-NH for pre-frailty and frailty. AUC = 0.805, *P* < 0.001. **b** Receiver operating characteristic (ROC) curves of FRAIL-NH for pairwise comparisons (The gold standard FI: FI ≤ 0.10 for non-frailty, 0.10 < FI ≤ 0.21 for pre-frailty, and FI > 0.21 for frailty; FRAIL-NH: FRAIL-NH = 0 for non-frailty, 0 < FRAIL-NH ≤ 4 for pre-frailty, and FRAIL-NH > 4 for frailty). 1: ROC Curve of FRAIL-NH for non-frailty and pre-frailty, AUC = 0.764, *P* < 0.001. 2: ROC Curve of FRAIL-NH for non-frailty and frailty, AUC = 0.910, *P* < 0.001. 3: ROC Curve of FRAIL-NH for pre-frailty and frailty, AUC = 0.781, *P* < 0.001. **c** Receiver operating characteristic (ROC) curves of FRAIL-NH for pairwise comparisons (The gold standard FI: FI ≤ 0.20 for non-frailty, 0.20 < FI < 0.45 for pre-frailty, and FI ≥ 0.45 for frailty; FRAIL-NH: FRAIL-NH ≤ 1 for non-frailty, 1 < FRAIL-NH ≤ 9 for pre-frailty, and FRAIL-NH > 9 for frailty). 1: ROC Curve of FRAIL-NH for non-frailty and pre-frailty AUC = 0.820, *P* < 0.001. 2: ROC Curve of FRAIL-NH for non-frailty and frailty, AUC = 0.976, *P* < 0.001. 3: ROC Curve of FRAIL-NH for pre-frailty and frailty. AUC = 0.817, *P* < 0.001

To further analyze the discriminative ability of FRAIL-NH between each pair of frailty classes in three-class frailty, a pairwise post-hoc test was conducted. The ROC curves of each pair were shown in Fig. 1a-c. When the cutoff points of the FI developed by Song et al. [28] were considered, the areas under the three pairwise ROC curves (Fig. 1a) were 0.819 for the curve corresponding to the comparison between subjects with non-frailty versus pre-frailty, 0.963 for the comparison between non-frail individuals and frail individuals, and 0.805 for the comparison between pre-frail individuals versus subjects with frailty.

When the cutoff points of the FI constructed by Hoover et al. [5, 27] were considered, the areas under the three pairwise ROC curves (Fig. 1b) were 0.764 for the curve corresponding to the comparison between subjects with non-frailty versus pre-frailty, 0.910 for the comparison between non-frail individuals and frail individuals, and 0.781 for the comparison between pre-frail individuals versus subjects with frailty. When the cutoff points of the FI created by Saum et al. [29] were considered, the areas under the three pairwise ROC curves (Fig. 1c) were 0.820 for the curve corresponding to the comparison between subjects with non-frailty versus pre-frailty, 0.976 for the comparison between non-frail individuals and frail individuals, and 0.817 for the comparison between pre-frail individuals versus subjects with frailty.

Table 4 shows the agreement in classification for two-class frailty between FRAIL-NH and the FI. The Kappa agreement of two categories of these two frailty measures was 0.529 (*P* < 0.001), 0.551 (*P* < 0.001) and 0.330 (*P* < 0.001) when using a FI cutoff point of 0.25, 0.21 and 0.45, respectively. It was observed that when the FI cutoff points of 0.21 and the FRAIL-NH cutoff points of 2 were used for frailty, the agreement value of two classes of these measures was at a maximum, 80.5% ([67 + 176]/302). Based on these cutoff points, the prevalence of frailty was 66.6% according to the FI and 69.5% according to the FRAIL-NH.

**Table 4:** The agreement in classification for two-class frailty between FRAIL-NH and FI

FRAIL-NH, n (%)	FI, n (%)		Total, n (%)	Kappa	*P* value
	Non-frail + Pre-frail (FI < 0.25)	Frail (FI ≥ 0.25)			
Non-frail + Pre-frail (FRAIL-NH < 4)	110 (90.2)	61 (33.9)	171 (56.6)	0.529	<0.001
Frail (FRAIL-NH ≥ 4)	12 (9.8)	119 (66.1)	131 (43.4)		
Total, n (%)	122 (40.4)	180 (59.6)	302 (100)		
	Non-frail + Pre-frail (FI ≤ 0.21)	Frail (FI > 0.21)			
Non-frail + Pre-frail (FRAIL-NH < 2)	67 (66.3)	25 (12.4)	92 (30.5)	0.551	<0.001
Frail (FRAIL-NH ≥ 2)	34 (33.4)	176 (87.6)	210 (69.5)		
Total, n (%)	101 (33.4)	201 (66.6)	302 (100)		
	Non-frail + Pre-frail (FI < 0.45)	Frail (FI ≥ 0.45)			
Non-frail + Pre-frail (FRAIL-NH < 8)	236 (82.8)	1 (5.9)	237 (78.5)	0.330	<0.001
Frail (FRAIL-NH ≥ 8)	49 (17.2)	16 (94.1)	65 (21.5)		
Total, n (%)	285 (94.4)	17 (5.6)	302 (100)		

*FI* Frailty Index

Table 5 presents the agreement in classification for three-class frailty between FRAIL-NH and the FI. The Kappa agreement of three classes of both frailty measures was 0.404 (*P* < 0.001), 0.350 (*P* < 0.001) and 0.445 (*P* < 0.001) when the cutoff points of the FI suggested by Song et al. [28], Hoover et al. [5, 27], and Saum et al. [29] were used, respectively. When the FI cutoff points of 0.20 and 0.45, and the FRAIL-NH cutoff points of 0 and four were used, the agreement value of three classes of both frailty measures was at a maximum, with 70.2% ([61 + 139 + 12]/302). A total of 180 (59.6%), 201 (66.6%) and 17 (5.6%) individuals were classified as frail by the FI suggested by Song et al. [28], Hoover et al. [5, 27], and Saum et al. [29], respectively. However, 41.1, 44.8 and 29.4% of those were misclassified as pre-frailty by FRAIL-NH, respectively. Based on the largest agreement, the prevalence of pre-frailty and frailty measured by FI were 65.9 and 5.6%, respectively; those measured by FRAIL-NH were 56 and 13.6%, respectively.

**Table 5:** The agreement in classification for three-class frailty between FRAIL-NH and FI

FRAIL-NH, n (%)	FI, n (%)			Total, n (%)	Kappa	*P* value
	Non-frail (FI ≤ 0.08)	Pre-frail (0.08 < FI < 0.25)	Frail (FI ≥ 0.25)			
Non-frail (FRAIL-NH = 0)	8 (80.0)	26 (23.2)	4 (2.2)	38 (12.6)	0.404	<0.001
Pre-frail (0 < FRAIL-NH ≤ 4)	2 (20.0)	85 (75.9)	74 (41.1)	161 (53.3)		
Frail (FRAIL-NH > 4)	0 (0.0)	1 (9.0)	102 (56.7)	103 (34.1)		
Total, n (%)	10 (3.3)	112 (37.1)	180 (59.6)	302 (100)		
	Non-frail (FI ≤ 0.10)	Pre-frail (0.10 < FI ≤ 0.21)	Frail (FI > 0.21)			
Non-frail (FRAIL-NH = 0)	13 (65.0)	17 (21.0)	8 (4.0)	38 (12.6)	0.350	<0.001
Pre-frail (0 < FRAIL-NH ≤ 4)	7 (35.0)	64 (79.0)	90 (44.8)	161 (53.3)		
Frail (FRAIL-NH > 4)	0 (0.0)	0 (0.0)	103 (51.2)	103 (34.1)		
Total, n (%)	20 (6.6)	81 (26.8)	201 (66.6)	302 (100)		
	Non-frail (FI ≤ 0.20)	Pre-frail (0.20 < FI < 0.45)	Frail (FI ≥ 0.45)			
Non-frail (FRAIL-NH ≤ 1)	61 (70.9)	31 (15.6)	0 (0.0)	92 (30.5)	0.445	<0.001
Pre-frail (1 < FRAIL-NH ≤ 9)	25 (29.1)	139 (69.8)	5 (29.4)	169 (56.0)		
Frail (FRAIL-NH > 9)	0 (0.0)	29 (14.6)	12 (70.6)	41 (13.6)		
Total, n (%)	86 (28.5)	199 (65.9)	17 (5.6)	302 (100)		

*FI* Frailty Index

## Discussion

To the best of our knowledge, this is the first study to assess the discriminatory performance of FRAIL-NH and to examine the agreement between FRAIL-NH and FI among nursing homes in mainland China, considering two-class and three-class frailty at the same time. Our study found that frailty could be identified by the 9 < FRAIL-NH ≤ 14, or 0.45 ≤ FI ≤ 1; pre-frailty could be identified by 1 < FRAIL-NH ≤ 9, or 0.20 < FI < 0.45. FRAIL-NH is a useful tool for frailty screening in Chinese nursing homes; however, it can be used only for preliminary screening because of moderate discrimination between adjacent two frailty classes. The level of agreement between FRAIL-NH and FI ranged from fair to moderate due to their large heterogeneity of captured domains and assessment items. Besides, it is notable that the cutoff points of FRAIL-NH for frailty and the prevalence of frailty could vary depending on the number of classifications.

The results from the three-way ROC analysis showed that whatever FI cutoff points were based on, the VUS values for FRAIL-NH—even the lower limit of the 95% CI of each VUS—were higher than the VUS of a useless test (1/6). This indicates that FRAIL-NH is a useful tool for frailty screening in Chinese nursing homes. This indication can also be drawn from the AUC values for FRAIL-NH that were obtained in two-way ROC analysis because these values were higher than the clinically meaningless value (0.5) and represented moderate to high discriminative accuracy for FRAIL-NH. Relatively, when the cutoff points of the FI developed by Saum et al. [29] were used, the FRAIL-NH cutoff points of 1 and 9 in classifying three-class frailty had the highest VUS and the largest CCRs. Therefore, the FRAIL-NH cutoff points of 1 and 9 may be the most appropriate to use for categorizing non-frailty, pre-frailty and frailty in Chinese nursing homes. Similarly, the FRAIL-NH cutoff point of 8 in classifying two-class frailty had the highest sensitivity and relatively high specificity. Therefore, it may be the most appropriate to use in Chinese nursing homes when a two-class situation is taken into account.

It should be noted that these FRAIL-NH cutoff points determined in this study need to be further tested in cohort studies. Besides, according to the cutoff points of the FI developed by Song et al. [28] and Hoover et al. [5, 27], the pair of FRAIL-NH cutoff points established in this study were 0 and four, which were consistent with those determined in 2380 nursing home older adults in Hong Kong, based on the suggested cutoff points of activities of daily living (ADL) [13]. Although the reference standard in this study was different from that in our study, the cutoff points of FRAIL-NH determined in those two studies were still the same. This may be because there were some overlaps in measurement items between FI and ADL. Besides, the cultural and ethnic similarity between mainland China and Hong Kong may be another reason because frailty thresholds vary in different cultures and ethnicities [15]. The mean FRAIL-NH and FI scores obtained in our study were lower than those in a Australian study (4.7 ± 4.1 and 0.35 ± 0.13, respectively) [7]. In that study, aged care facility residents aged 65 and older (mean age 87.5) were investigated, which is likely to contribute to the higher frail level. A few studies have proved that the level of frailty increases with age [4, 5, 22]. We should be cautious, however, to compare this result because of the slight differences between the contents of FRAIL-NH and FI in our study and those in previous studies [7, 8, 11, 13].

The three-way ROC analysis gave a single measure for the overall performance of FRAIL-NH and a set of cutoff points for the whole classes [32]. It cannot assess the discriminative performance of each pair of frailty classes. However, sometimes, the analyses between different sub-groups are necessary because they can provide useful information when classifying individuals. Through post hoc pairwise comparison, we found that the performance of FRAIL-NH in distinguishing between pre-frailty and frailty is the same as when distinguishing between non-frailty and pre-frailty, although its discriminatory ability was moderate to high in distinguishing between each pair of frailty classes. As expected, FRAIL-NH had a higher performance when distinguishing between non-frailty and frailty. That is to say, FRAIL-NH was moderately discriminating between two adjacent frailty classes. Therefore, FRAIL-NH can be used to preliminarily identify people with possible non-frailty and frailty as the first step in a two-stage assessment in nursing home settings or as a quick screening tool integrated into routine nursing home practice because of its brevity and low-assessment burden feature. It is worth noting that more work needs to be done to verify such findings since fewer numbers of people in the group with non-frailty in this study may impose additional restrictions on sub-group analyses. A larger sample size is required in sub-groups to conduct similar analyses.

In this study, the agreement between FRAIL-NH and FI ranged from fair to moderate, regardless of what cutoff points were used. This is likely because there was large heterogeneity in captured domains (see Additional file 1: Tables S1 and S2) and assessment items (7 vs. 34) between these two measures. Co-morbidities, cognition, mood, nutrition, physical function, continence, and polypharmacy are the broad domains covered by the FI. However, the FRAIL-NH does not include cognition. Additionally, the FRAIL-NH is more aligned with the phenotypic variables [12]. Therefore, the agreement of FRAIL-NH with FI was not that good. In clinical practice, individuals who are frail but misclassified as pre-frail should receive more attention as frail older adults need timely intervention [32]. In this study, according to FRAIL-NH, the proportion of frail individuals who were misclassified as pre-frail was higher than that of non-frail individuals misclassified as pre-frail, whichever FI comparators were chosen. Therefore, to avoid frail individuals being neglected, it would be necessary to further assess frailty status by the FI, especially for those classified as pre-frailty by FRAIL-NH.

Surprisingly, our study showed that when the same FI was used as a reference, the FRAIL-NH cutoff points for two-class frailty were different from those for three-class frailty.

For example, based on the FI cutoff points developed by Saum et al. [29], the FRAIL-NH cutoff point for frailty was 8 when two-class frailty was considered, while the corresponding cutoff point was 9 in the three-class situation. Additionally, the corresponding prevalence of frailty also differed (21.5% VS 13.6%), even when using the same measures in the same population. These findings suggest that the cutoff points of FRAIL-NH for frailty as well as the prevalence of frailty could vary depending on the number of classifications. Therefore, in clinical and research practice, it is not advisable to arbitrarily combine two adjacent classes to form one class, because it may lead to the loss of some information.

Because different frailty measures and cutoff points were used in this study, the prevalence of pre-frailty and frailty varied (26.8 to 65.9%; 5.6 to 69.5%, respectively). This is similar to what was reported in a recent systematic review (pre-frailty: 28.9–52.1%; frailty: 37.9–66.5%) [4]. Additionally, by comparing the prevalence rates measured by FRAIL-NH and FI, we found that FI tended to classify individuals as frail, whereas FRAIL-NH tended to classify individuals as pre-frail. This could be due to the fact that, compared to FRAIL-NH, FI included multidimensional health deficits, such as psychology and cognition. Therefore, FI could categorize people who were at risk of frail while FRAIL-NH categorizes them as pre-frail.

There are several limitations that should be noted. Due to limited research conditions, we only investigated residents living in large-scale nursing homes in Changsha, a typical second-tier city in China’s central region with a specific regional representation [34, 35]. Therefore, FRAIL-NH cutoff points determined in the current study should be cautiously applied. The study’s generalizability may be limited if these cutoff points were applied to small/medium-scale nursing homes or nursing homes in cities with populations with different socioeconomic status. In addition, this study used convenience sampling, which may limit the representativeness of the research samples. Moreover, the sample size should be expanded to further verify the current findings in future studies. Finally, the fact that no health outcomes were evaluated to guide the selection of cutoff points is also a limitation of this study.

## Conclusions

FRAIL-NH can be used as a preliminary frailty screening tool in nursing homes in mainland China. FI should be further used to determine frailty status if individualized interventions need to be designed and implemented in nursing home older adults, especially among those classified as pre-frailty by FRAIL-NH. It is not advisable to simply combine two adjacent classes of FRAIL-NH to form a new frailty variable in research settings. The FRAIL-NH cutoff points determined in this study need to be further tested in future research, and findings in this study also need to be verified in larger studies.

## Supplementary information

**Additional file 1: Table S1.** The FRAIL-NH scale. **Table S2.** The items and coding of Frailty Index.

## Data Availability

The datasets used for the current study are available from the corresponding author upon reasonable request.

## Abbreviations

AUC: the area under the ROC curve
BMI: body mass index
CCR: correct classification rates
CI: confidence interval
FI: Frailty Index
PPF: Physical Frailty Phenotype
ROC: receiver operating characteristic curves
SD: standard deviation
SE: standard error
TFI: Tilburg Frailty Indicator
VUS: volume under the ROC surface
