# Supplementary material for: Accessing the discriminatory performance of FRAIL-NH in two-class and three-class frailty and examining its agreement with the frailty index among nursing home residents in mainland China
Source: BMC Geriatr. 2019 Oct 30;19:296. doi: 10.1186/s12877-019-1314-9 (PMC6822433; doi:10.1186/s12877-019-1314-9)
Supplement: Supplementary file 1 — Additional file 1: Table S1. The FRAIL-NH scale. Table S2. The items and coding of Frailty Index. [file 12877_2019_1314_MOESM1_ESM.docx]

**Additional file 1**

Table S1. The items and coding of Frailty Index

| Hypertension (1=yes, 0=no) | Obesity (BMI)  ( ≥ 28=1, 24.0～27.9=0.5, 18.5～23.9=0) |
| --- | --- |
| Chronic renal failure (1=yes, 0=no) | Constipation (1=yes, 0=no) |
| Chronic obstructive pulmonary disease  (1=yes, 0=no) | Pressure ulcers (1=yes, 0=no) |
| Heart failure (1=yes, 0=no) | Anemia (1=yes, 0=no) |
| Cancer (1=yes, 0=no) | Visual impairment (1=yes, 0=no) |
| Stroke (1=yes, 0=no) | Hearing impairment (1=yes, 0=no) |
| Parkinson (1=yes, 0=no) | Slow walking speed (1=yes, 0=no) |
| Atrial ﬁbrillation (1=yes, 0=no) | Falls (1=yes, 0=no) |
| Gastrointestinal problems (1=yes, 0=no) | Unintentional weight loss (1=yes, 0=no) |
| Thyroid disorders (1=yes, 0=no) | Difﬁculties taking a bath or shower (1=yes, 0=no) |
| Diabetes mellitus (1=yes, 0=no) | Dressing difﬁculties (1=yes, 0=no) |
| Psychiatric disease (1=yes, 0=no) | Difﬁculties with personal hygiene (1=yes, 0=no) |
| Gastrointestinal or liver disease (1=yes, 0=no) | Difﬁculties with transfer (1=yes, 0=no) |
| Musculoskeletal diseases (1=yes, 0=no) | Eating problems (1=yes, 0=no) |
| Depression (PHQ-9 ≥ 5) (1=yes, 0=no) | Difﬁculties with urinary incontinence (1=yes, 0=no) |
| Dementia (MMSE)  ( ≤ 14=1, 15～23=0.5, ≥ 24=0) | Difﬁculties with fecal incontinence (1=yes, 0=no) |
| Malnutrition (MNA-SF) ( <11=1, ≥ 11=0) | Polypharmacy ( ≥ 5) (1=yes, 0=no) |

*PHQ-9* The 9-item Patient Health Questionnaire Depression Scale; *MMSE* The Mini-Mental State Examination; *MNA-SF* The Short-form Mini-Nutritional Assessment Scale; *BMI* Body mass index (weight/height^2^, kg/m^2^)

Table S2. The FRAIL-NH scale

| Item |  | Level |  |
| --- | --- | --- | --- |
|  | 0 | 1 | 2 |
| Energy | Good/excellent | Fair | Poor |
| Transferring | Moves in and out of bed or chair unassisted. Mechanical transfer aids are acceptable | Needs help moving from bed to chair or requires complete transfer | Needs help in moving from bed to chair or requires complete transfer and Katz score <3 |
| Mobility | Goes out | Able to get out of bed or chair but does not go out | Bed or chair bound |
| Continence | Exercises complete self-control over urination and defecation | Partial or total bowel or bladder incontinence | Partial or total bowel or bladder incontinence and Katz score < 3 |
| Weight loss  (last 3 months) | No weight loss | 1-3 kg or does not know | > 3kg |
| Feeding | Gets food from a plate into the mouth without help. Preparation of food may be done by another person | Needs partial or total help with feeding or requires parental feeding | Needs partial or total help with feeding or requires parental feeding and Katz score <3 |
| Dressing | Gets clothes from closets and drawers and puts on clothes and outer garments complete with fasteners. May have help with tying shoes | Needs help with dressing self or needs to be completely dressed | Needs help with dressing self or needs to be completely dressed and Katz score <3 |
